# Reducing applied force in colonoscopy using a novel soft robotic colonoscope: Head-to-head study

**DOI:** 10.1055/a-2641-5827

**Published:** 2025-07-24

**Authors:** Jabed Foyez Ahmed, Korn Borvorntanajanya, Jialei Shi, Enrico Franco, Ara Darzi, Ferdinando Rodriguez Baena, Nisha Patel

**Affiliations:** 1 4615 The Hamlyn Centre for Robotic Surgery, Imperial College London, London, United Kingdom of Great Britain and Northern Ireland; 2 8946 Gastroenterology, Imperial College Healthcare NHS Trust, London, United Kingdom of Great Britain and Northern Ireland

**Keywords:** Endoscopy Lower GI Tract, Quality and logistical aspects, Training, Quality management, Performance and complications

## Abstract

**Background and study aims:**

Current colonoscopies have a recognized limitation. Manual pushing and pulling required by operators provides gross movement in the bowel. Reported pain, therefore, is likely due to the manual force applied. Implementing novel steering techniques with a soft growing robotic system can potentially overcome challenges such as fine control, precise steering, and capability to expand treatment options for complex therapies. This study assessed a novel controlled-growing soft robot compared with a standard colonoscope in terms of force exhibited on a model bowel wall and its clinical implications.

**Methods:**

A head-to-head study using a hybrid colon phantom of the left colon was undertaken. Both the novel soft-robot and standard colonoscope were passed through the phantom by endoscopists. Multiple passes were undertaken in the phantom with both colonoscopy methods with force values recorded at two points (rectum and sigmoid) with pressure sensors.

**Results:**

Nine clinical endoscopists (4M:5F, 5 non-expert, 4 expert) were recruited. Average force with the novel robot was 0.25N (rectum) and 0.19N (sigmoid). Average force applied with standard colonoscopy was 2.82N (rectum) and 1.45N (sigmoid).

**Conclusions:**

This study demonstrated an improvement in force with the novel soft robot compared with a standard colonoscope. This suggests the possibility of more comfortable colonoscopy for patients. Currently time taken is longer with the novel robot, which is attributable to the learning curve and improves in subsequent passes. Further work will be undertaken in a complete colon model with aspirations to reach in-vivo experiments.

## Introduction

Colonoscopy is the gold standard for diagnostic investigation and therapeutic intervention for the lower gastrointestinal tract 1 . Since its inception in 1806 by Bozzini 2 to its further development with fiber optic technology in 1957 by Hirschowitz 3 , the design and fundamental setup of the colonoscope has not changed. Ongoing image quality improvement and addition of instrument channels, suction, and inflation capabilities has allowed a higher-quality diagnostic procedure. Development of cables that allow tip deflection in 1970 4 along with video capabilities has resulted in a colonoscope very similar to the current version used today. 5

Therapy, while ever expanding with more complex procedures such as endoscopic submucosal dissection (ESD) and hybrid ESD, is limited to what is deliverable through the current existing endoscope. There is a recognized gap in capability of treatment options for pathology that is too advanced for current endoscopic techniques and too invasive for surgical intervention via a minimally invasive approach with endoscopy.

A limitation of the current colonoscope and consequent manual technique employed is the high potential to induce pain and discomfort in patients. Some factors causing discomfort are patient-specific, such as anatomy and pre-procedure anxiety. Other factors are a result of operator skill, such as level of experience, technique, torque steering, and total procedure time. These factors will directly influence the total pushing force applied and reporting of pain and discomfort 6 . Manual pushing and pulling does not allow a set defined force for advancement and withdrawal. It is entirely based on user fine motor control and touch. For complex and delicate interventions, this may be a limitation. This pushing technique also increases the learning curve for trainee endoscopists who need to learn to differentiate the tactile feedback to ensure they are making safe progression in the colon. Ergonomic strain may also occur for the endoscopist, which may have long-term musculoskeletal consequences. Another major limitation is steering capability of the standard colonoscope with a somewhat restricted stable platform, lack of bimanual control, and triangulation of instruments limiting its use in advanced therapeutics. This can be partially resolved with a two-person insertion and “three-hand technique”. However, lack of training in these modalities and inability to meet increased requirement of endoscopists may prevent these being implemented in real clinical practice.

Implementing a robotic system can potentially overcome these issues and allow fine control, precise steering, and capability to expand endoscopy treatment options further. This has been demonstrated in other medical fields and is slowly being introduced into the gastrointestinal and endoscopy landscape 7 . This does, however, come with a possibility of a new presentation of repetitive strain injuries such as gamers thumb and gamers grip 8 .

In the first instance, further high-quality training in tip control is the priority for trainee endoscopists and will aid in overcoming issues of fine control and precise steering. When this is not possible, implementing a robotic system can be a solution with the added capability of not only expanding treatment options available but reducing the difficulty and learning curve and improving procedure outcomes in endoscopy such as reported by Chiu et al using robotic systems in ESD 9 .

Potential benefits that robotic systems can bring to endoscopy include reduced cost of production, reduced or complete elimination of reprocessing 10 11 12 and cleaning of equipment, increased access in rural and low-income countries, and with the option of single-use systems, reduction in infection and procedure-related illnesses. Evidence about cost-effectiveness in robotic endoscopy is currently not available and an area of ongoing work to add evidence to its utility and potential benefit. Research and data from robotic surgery can be referred to as an evidence base for its potential translation to endoscopy 13 14 .

A knock-on consequence of cost reduction will be potential feasibility of implementation in rural areas where there is no infrastructure for reprocessing and also low-income countries with funding being viable for investment in endoscopy services due to these cost reductions. Further data are needed to create a stronger evidence base for this claim 15 .

Our long-term goal is to develop a new soft robotic endoscope capable of navigating hollow visceral organs through eversion, aiming to reduce patient discomfort during colonoscopy procedures. This paper presents experimental evaluation of a novel self-propelling prototype that employs the eversion principle.

Results of a head-to-head study using a hybrid soft and rigid colon phantom resembling the left colon sigmoid indicate that the new soft robotic system results in reduced contact forces compared with a standard colonoscope. This has a potential clinical consequence of a more comfortable procedure for patients and with better steerability and fine control. This may then allow expansion to develop treatment options in colonoscopy further.

## Methods

**Fig. 1:**
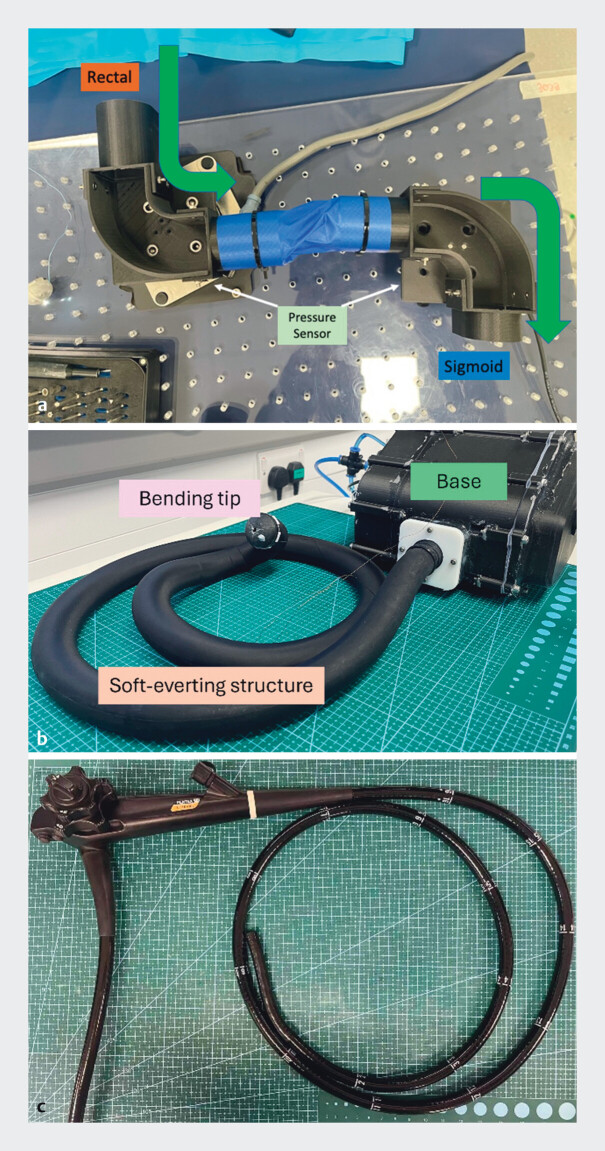
**a** Soft phantom setup (green arrow indicates direction of forward movement). **b** Soft growing robot. **c** Standard colonoscope.

**Fig. 2:**
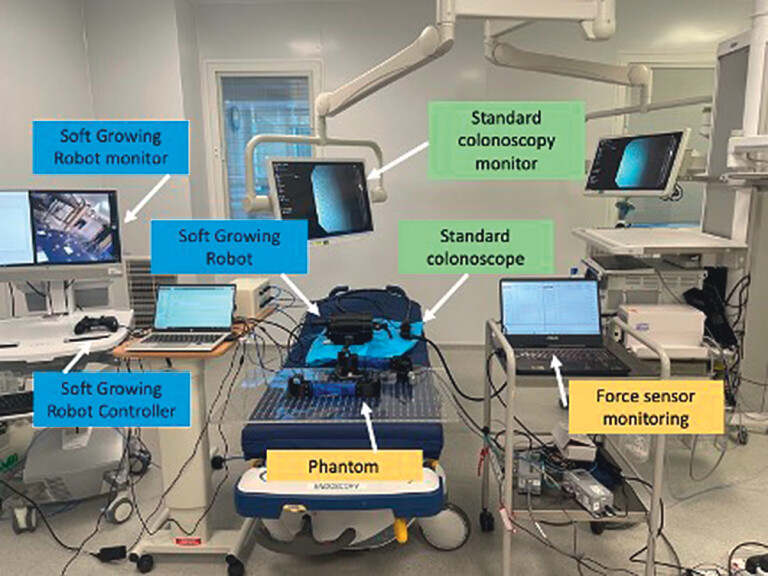
Bench top experiment setup.

**Fig. 3:**
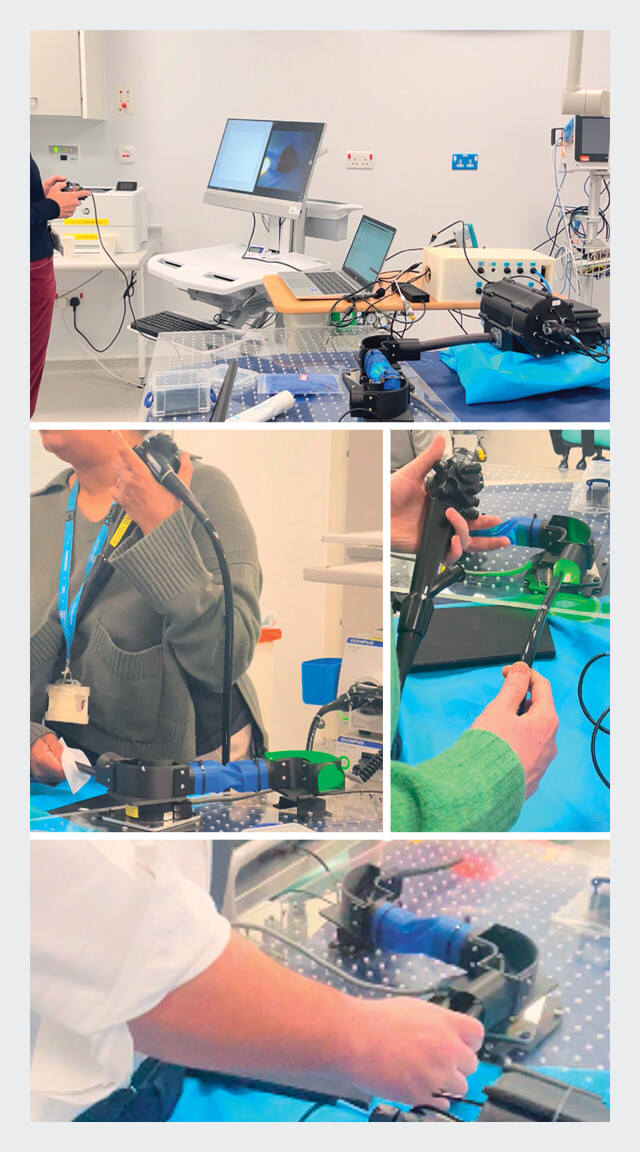
Endoscopist runs with Soft growing robot and standard colonoscopy.

This was a single-center study to assess standard colonoscopy versus novel soft robotic colonoscopy using a phantom model by clinical endoscopists. The study recruited nine endoscopists (5 consultants, 3 registrars, 1 nurse endoscopist). Each endoscopist performed colonoscopy four times (1 practice, 3 recorded trial run) on the phantom model. This was either standard colonoscopy first followed by novel soft robotic colonoscopy or vice versa ( Fig. 1 , Fig. 2 , Fig. 3 ).

Primary outcomes measured were force and time. Force comprised maximum and minimum pressure exhibited during the colon route in phantom, average pressure exhibited during the colon route, and comparison between standard and novel colonoscopes. Time comprised maximum and minimum time to completion of colon route in phantom, average time for completion of the colon phantom route, and comparison between standard and novel colonoscopes

Pressure sensors were placed below the rigid phantom areas of the colon model ( Fig. 1 **a** ). The phantom comprises two L-shaped corners connected by a complaint fabric tube and represents a section of the human sigmoid colon. Each corner has a width of 40 mm and a radius of 55 mm. The two rigid parts were fixed to two Force/Torque sensors (Gamma NANO, ATI) to measure interaction forces between the robot and the phantom during the operation.

### Data analysis

Force readings were collected from the two sensors placed on the rectal and sigmoid regions of the phantom and collected on MATLAB via ethernet port. Each run was recorded using handheld video to document timing alongside the timestamp on MATLAB of the phantom run also.

## Results

Nine endoscopists (4 male, 5 female), with an average modal age range between 35 and 44 years with a varying range of endoscopist experience ( **Supplementary Fig. 1** ) were recruited for the head-to-head study.

Endoscopy experience ( **Supplementary Fig. 2** , **Supplementary Fig. 3** ) varied from expert to novice over a period < 5 years to > 20 years.

### Force results

Average force applied with the novel robot to the end of rectum was 0.25N and to the end of the sigmoid was 0.19N. Average force applied with the standard colonoscopy to the end of the rectum was 2.82N and to the end of the sigmoid was 1.45N.

**Fig. 4:**
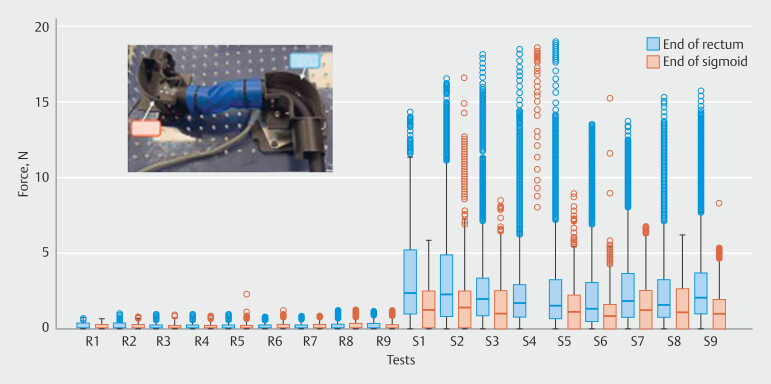
Force recorded summary for endoscopists in rectum (R) and sigmoid (S).

Maximum force applied with the novel robot to the end of the rectum was 1.31N and to the end of the sigmoid was 1.35N (SD: 0.18). Maximal force applied with the standard colonoscopy to the end of the rectum was 19.03N (SD: 2.79) and to the end of the sigmoid 18.6N (SD: 1.38) ( Fig. 4 ).

### Time results

Average time to complete the phantom with the standard colonoscopy was 32.38 seconds (SD: 4.53). Average time to complete the phantom with the novel robot was 59.17 seconds (SD 17.72).

### Endoscopist questionnaire

**Fig. 5:**
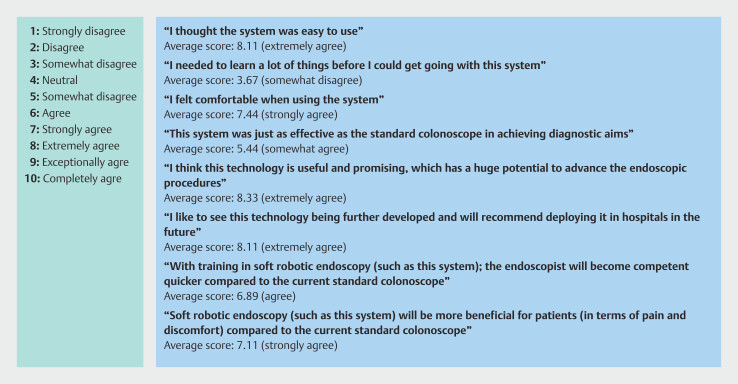
**a** Ten-point Likert scale of agreeability used for endoscopist questionnaire. **b** Questionnaire statements with average Likert score and statement.

The nine endoscopists were asked about their prior experience using a joystick/game controller in both clinical and non-clinical settings such as gaming. Seven of nine endoscopists (77.8%) had prior experience. Two of nine endoscopists (22.2%) also reported prior experience with robotic colonoscopy. The endoscopists answered the questions shown in Fig. 5 **a** using a 10-point Likert rating scale of agreeability to the statements. Fig. 5 **b** shows the average score for each statement.

### Head-to-head summary

Table 1 highlights key characteristics of the soft growing robot versus the standard colonoscope.

**Table:** **Table 1** Summary of characteristics and features for standard colonoscope vs soft growing robot.

	**Standard colonoscope**	**Novel soft robot colonoscope**
Width	1.0–1.2 cm	1.8 cm
Length	160–180 cm	160 cm
Degrees of freedom	2 DOF	3 DOF
Maximum force	19.03 Newtons	2.82 Newtons
Control	Pulley system controlled with fingers	Controller pad controlled with pressing buttons
Image	Standard definition (SD) 100,000–400,000pixels up to high definition (HD) 1 million pixels	160,000 pixels
Therapeutic potential	Large and advanced polypectomy with EMR and ESD Dilatation and APC and gastrointestinal bleed management	
APC, argon plasma coagulation; DOF, degrees of freedom; EMR, endoscopic mucosal resection; ESD, endoscopic submucosal dissection.		

## Discussion

This head-to-head study has demonstrated an exciting new method in the approach to performing colonoscopy and addressing issues of pain and discomfort experienced by patients and improving fine control for steering and potential therapeutic applications.

The salient findings from this study include the significant reduction in force applied with the robotic colonoscopy system. This has clinical implications of a more comfortable procedure for the patient and may help improve loss to follow up and increase engagement of patients who do not attend due to fear of pain 16 .

Total procedure time for the soft growing robot was longer than for the current standard colonoscopy procedure. However, with repeated runs, reduced total procedure time was demonstrated by the endoscopists. This suggests the possibility of achieving non-inferiority with the novel technique. This is important, because it will allow current service provision in place for standard colonoscopy to continue and allow no decrease in service output, which is an important metric on which endoscopy units are being evaluated, especially given the additional backlog created by the COVID-19 pandemic 17 .

Use of a gamepad controller allows ergonomic advantages indirectly because operators may assume a sitting position, reducing musculoskeletal risks associated with long periods of standing 18 . Controllers will also remove the hand span factor of operators, which can, in some instances, limit wheel movement 19 . New presentations of repetitive strain injuries such as gamers thumb and gamers grip, however, are a new consequence of performing repeated procedures with a gamepad, as seen in the professional gaming sector of Esports 8 .

From a learning curve perspective, the ability to achieve competency is potentially made quicker with endoscopist opinion reporting a controller setup is easier for learning control and steering. This was noted based on observing each endoscopist performing the run in the phantom. Having not undertaken any prior robotic colonoscopy steering with a controller, it was noted the improvement in time taken and force applied in as little as the four runs undertaken. In part, gamification also plays a role in popularity of gaming consoles in various user groups, including clinical endoscopists, and of a similar controller setup in the gaming setup. There was a generational observation made, wherein the younger cohort of endoscopists reported more familiarity and ease with the controller setup, which again reflect the gamification of technology 20 .

Minimal force measurements decrease risk of mucosal damage and the major complication of perforation to as insignificant a level as possible. The result, therefore, would be a safer procedure for all patients undertaking colonoscopy 21 . Barotrauma from increased air insufflation can be reported in up to 35% of cases 22 , whereas the minimal air pressure of 0.2 bar used in the soft growing robot compared with the 0.5bar in conventional endoscopy ensures that risk is not increased with this new technology 23 24 .

Some limitations observed included use of the hybrid soft and rigid phantom. It was noted due to the friction experienced with both the standard colonoscope and soft growing robot and may result in moments of the scope getting temporarily stuck. Subsequent “slipping” of the scope would then occur when the scope would move forward at an increased rate due to overcoming of the friction. Clinically this would require the scope to be reversed to ensure mucosa has been adequately assessed. With continued development of soft phantoms and compositions as close to the in vivo setting of a human colon, this is an area that will continue to improve to allow as close to clinical condition testing of new technology.

Loss of tactile feedback was the most common response obtained from the clinical endoscopists undertaking the procedure with the soft growing robot. This is a major change from the fundamental principle applied to the current technique, where resistance, torque, and feel play a role in achieving successful completion of a procedure and navigating challenges along the way, such as looping and negotiating acute bends in the colon 25 . This is further supported with the visual aid produced from magnetic endoscopy images such as ScopeGuide 26 . Removal of tactile feedback will take time to adjust, with learning of operators likely moving from unconscious competence to conscious competence at a slower rate in comparison 27 . This may not be the case for novice trainees who have no previous experience with the standard colonoscopy modality.

The soft growing robot is under active continued improvement along its development course. Some of the hurdles already overcome include refining the fine steering, which was achieved with a multi-pocket composition of the soft growing robot. Air leakage from multiple repeated use was overcome by using a more durable material.

Consideration of a one-time use option for each run of the soft growing robot in a clinical setting is being reviewed, given use of an inexpensive material and elimination of potential reprocessing costs. The drawback on balance will be the increased wastage from the one-time use material. This has been reflected in the ESGE recommendations about reducing the environmental footprint of gastrointestinal endoscopy in which single-use colonoscopy is not recommended routinely but rather, for specific cases only 28 .

Particular aims for future work on this project include developing the soft growing robot and conducting further head-to-head testing. Robot development will be undertaken by improving the responsiveness of the soft growing robot. Incorporating a working channel for biopsies is an essential requirement and also needed for the aspirational aims for its further development into therapeutic capabilities alongside improvement of the optical camera 29 .

Further head-to-head comparable testing with a standard colonoscopy will be undertaken. Performance of further bench-top testing in a full colon phantom setup of human length will enable clearer clinical correlation. Assessment of the capability of the soft growing robot to avoid looping and manage proximal and distal force difficulties will be of interest because currently, these results are unknown. Further improving the material used in the soft phantom to get as close to an in-vivo environment is also beneficial because it will allow a better clinical extrapolation and interpretation of forces and its role in reducing pain and discomfort in colonoscopy. Recruiting a larger number of clinical endoscopists will improve the power of the study, as would adding surgical trainees and consultants who perform colonoscopy. We also an aim to include all specialty endoscopists who undertake colonoscopy to form the best generalized assessment and conclusion.

## Conclusions

This study demonstrated that the novel soft robot colonoscope has the ability to significantly improve force applied in the colon for both average and single maximal value readings, which have a clinical impact on significantly more comfortable colonoscopy for patient, as is partially explained by reduced tension in the colonic ligaments.

Further benefits with this new technology include reducing or eliminating the need for sedation, better patient engagement with follow up, and an easier learning curve for endoscopists with earlier achievement of competence.

Currently time taken is longer with the novel robot and this can mainly be attributed to the learning curve for the new technology, but improvement can already be seen in subsequent passes, indicating a shallower learning curve.

Further work is planned to collect data in a complete soft phantom of an entire colon model with the aspirational aim of then performing animal model testing. Work on the composition of the phantom to achieve as close to in-vivo human characteristics is also ongoing. Widening the pool for recruitment and increasing the number of endoscopists will enable a better understanding of this new soft robot technology and its potentially beneficial impact.

## Supplementary Material

Supplementary Material Zusatzmaterial
