# Supplementary material for: Reducing applied force in colonoscopy using a novel soft robotic colonoscope: Head-to-head study
Source: Endosc Int Open. 2025 Jul 24;13:a26415827. doi: 10.1055/a-2641-5827 (PMC12372447; doi:10.1055/a-2641-5827)

Supplementary Figure 1: Endoscopist age range (years)

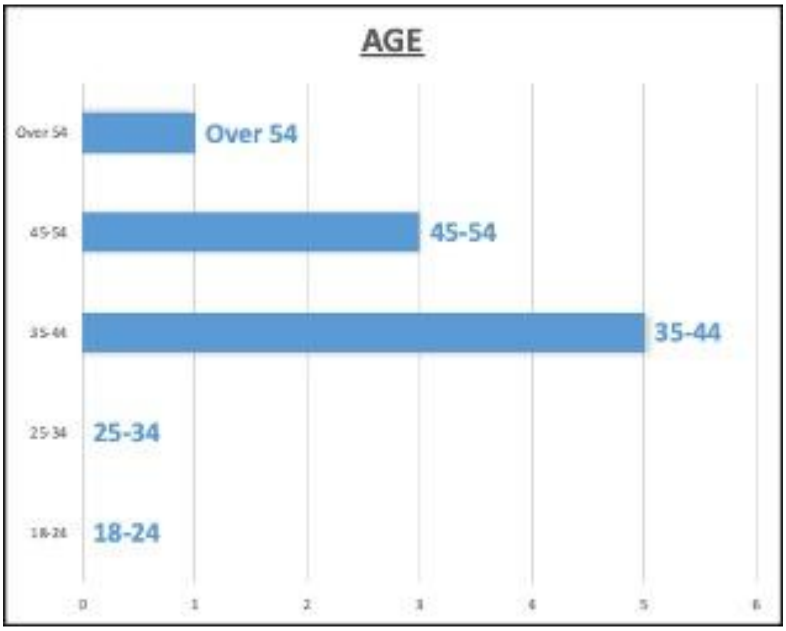

Supplementary Figure 2: Endoscopist experience (years)

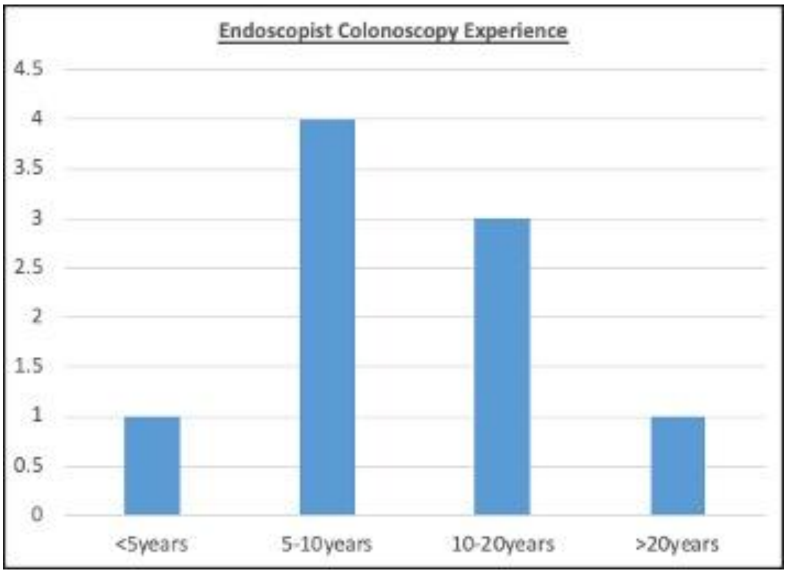

Supplementary Figure 3: Endoscopist experience (no. of procedures)

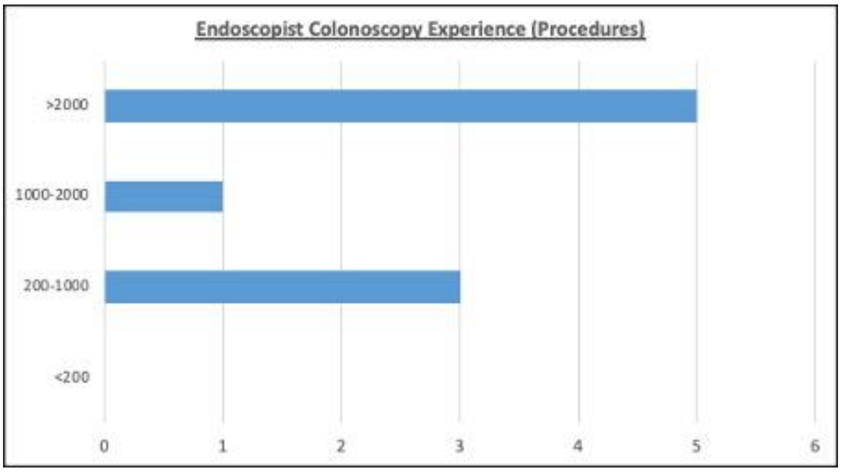

Supplement: Supplementary file 1 — Supplementary Material [file 10-1055-a-2641-5827_26513415.pdf]
